# Opioid and Nitrergic Pathways Mediate Antinociception by *Pectis elongata* Kunth. Essential Oil

**DOI:** 10.1002/cbdv.202501962

**Published:** 2025-10-22

**Authors:** Angela Palma Amorin, Sandra Layse Ferreira Sarrazin, Leomara Andrade da Silva, Thaiana Neves Coelho, Adriele Pantoja Cunha, Vitória Albuquerque de Lima, Domingas Machado da Silva, Pablo Luis Baia Figueiredo, Ricardo Bezerra de Oliveira

**Affiliations:** ^1^ Programa De Pós‐Graduação em Ciências da Saúde Universidade Federal do Oeste do Pará Santarem Brazil; ^2^ Programa De Pós‐Graduação em Biodiversidade e Biotecnologia Universidade Federal do Oeste do Pará Santarem Brazil; ^3^ Programa De Ciências Naturais, Instituto de Ciências da Educação Universidade Federal do Oeste do Pará Santarem Brazil; ^4^ Programa De Pós‐Graduação em Ciências Farmacêuticas Universidade Federal do Pará Belem Brazil

**Keywords:** citral, essential oil, inflammation, mechanism of action, pain

## Abstract

In the present study, we aimed to evaluate the antinociceptive potential of the *Pectis elongata* essential oil (PeEO) and to understand its mechanism of action by identifying possible pain inhibition pathways. PeEO was analyzed using gas chromatography coupled with mass spectrometry. Male Swiss mice were subjected to the hot plate test to assess analgesic activity, determine the effective dose, and elucidate the pharmacological pathways involved, using specific blockers of the opioid, cholinergic, and nitric oxide pathways. Chemical analysis identified 23 compounds accounting for 95.57% of the sample, with citral (86.4%—Geranial and Neral) as the major constituent. The antinociceptive activity of PeEO was observed at a dose of 600 mg/kg, and this effect was mediated by the opioid and nitrergic pathways, as evidenced by pretreatment with L‐NAME and naloxone. These findings reinforce the analgesic potential of PeEO, possibly related to its high citral content.

## Graphical abstract

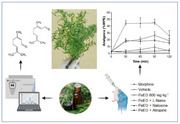

## 1 Introduction

Pain is considered an unpleasant sensory and emotional experience, often associated with or compared to actual or potential tissue damage [1]. It is a personal experience, shaped by biological, psychological, and social factors [2]. According to the latest Global Pain Index (GPI) report, up to 93% of people worldwide suffer from physical and mental pain, with one‐third experiencing pain daily. Brazil ranks fourth among the 19 countries studied, with 97% of the population suffering from pain episodes.

To relieve the discomfort caused by pain, analgesic medications are used that interfere with the production of nociceptive impulses in the nervous system, either peripherally or centrally [3]. Among the classes of drugs used for pain relief are opioid receptor agonists, non‐steroidal anti‐inflammatory drugs (NSAIDs), tricyclic antidepressants (TCAs), N‐methyl‐D‐aspartate (NMDA) receptor antagonists, adrenergic agonists, and antiepileptic drugs (sodium channel blockers) [4].

Although these medications are essential in pain management, they can cause a range of adverse reactions [5, 6], and despite a better understanding of the molecular mechanisms of nociception, existing analgesics remain limited in terms of efficacy in chronic conditions [7]. Therefore, considering the side effects and the reduction in antinociceptive efficacy observed in long‐term treatments, it is necessary to explore alternative or adjunctive medications for pain management.

In this context, medicinal plants, with their natural properties, emerge as a viable alternative, reducing the need for multiple treatments and the burden of side effects. Throughout history, various cultures have used them for pain treatment [8, 9], and different studies validate their therapeutic properties, highlighting them as a safe and effective option [10].

In the northern region of Brazil, it is common for riverine, quilombola, and indigenous populations to use medicinal plants to treat diseases, due to easy access to these natural resources and the difficulty in accessing urban areas [11]. *Pectis elongata* Kunth. (Asteraceae), a plant native to northern Brazil has demonstrated significant pharmacological potential. In the state of Pará, this species is popularly known as “cominho” or “limãozinho”. It is a species cultivated and sold at open markets in Santarém, Pará, for both food and medicinal use.

*P. elongata* is an aromatic species, rich in essential oil (EO), with two chemotypes identified: citral [12, 13, 14, 15] and perillaldehyde [16]. Of these, citral has stood out for its analgesic and anti‐inflammatory properties [17, 18]. However, few studies have revealed the possible mechanism of action involved. In the present study, we aimed to evaluate the antinociceptive potential of the EO of *P. elongata* (PeEO) and understand its mechanism of action, determining the possible pain inhibition pathways used by this EO. Finally, we highlight that the search for new potential analgesic drugs is not only a scientific advancement but also a necessary response to the limitations of current treatments, with prospects for safer and more effective therapies for pain management.

## 2 Results and Discussion

### 2.1 Yield and Chemical Composition of EO

The yield of PeEO was determined to be 1.5%. In the analysis of the chemical composition, 23 components were identified, representing 95.57% of the total. Of these, geranial (49.61%) and neral (36.77%) were the major constituents (Table 1). Together, these stereoisomers form citral. These results corroborate the findings of [15], who also identified citral as the main compound in samples of *P. elongata* collected in Santarém, Pará, although their yield was only 1%. In contrast, the study by [13] found a PeEO yield of 2.5%.

**TABLE 1:** Chemical constituents present in the essential oil of *Pectis elongata*.

Peaks	RT	Chemicals constituents	PeEO (%)
1	7.024	6‐methyl‐5‐hepten‐2‐one	0.27
2	7.639	NI	0.03
3	8.282	*p*‐cimene	0.02
4	8.426	Limonene	0.23
5	10.663	NI	0.08
6	10.990	Linalool	0.29
7	12.785	(exo) Isocitral	0.25
8	13.125	Citronellal	0.07
9	13.601	(Z) Isocitral	0.89
10	14.058	Rosefuran epoxide	0.11
11	14.244	NI	0.08
12	14.357	(E) Isocitral	1.76
13	14.737	α‐Terpineol	0.03
14	14.858	NI	0.02
15	15.133	cis‐Piperitol	0.03
16	15.389	NI	0.03
17	16.088	Citronellol	0.16
18	16.340	Nerol	0.44
19	16.973	Neral	36.77
20	17.455	Geraniol	0.89
21	18.296	Geranial	49.61
22	18.543	NI	1.25
23	19.030	(1)Tridecene	1.81
24	20.952	NI	0.79
25	21.803	Geranic acid	0.36
26	21.968	NI	0.32
27	22.484	NI	1.37
28	23.268	*β‐*elemene	0.23
29	23.578	Cyperene	0.11
30	23.729	NI	0.07
31	24.630	NI	0.10
32	24.719	NI	0.14
33	24.810	NI	0.06
34	25.774	α‐ Humulene	0.72
35	27.325	Dodecanol	0.06
36	31.872	Humulene epoxide II	0.46
37	41.856	NI	0.03
38	42.010	NI	0.06
Total identified constituents	95.6%		

RT—Retention Time; NI—Not Identified; PeEO—*Pectis elongata* essential oil.

Previous studies report the existence of two distinct chemotypes for the species, identified as perilla aldehyde and citral. Silva et al., for example, observed perilla aldehyde as the major compound in samples collected in Pará and Amapá. Massing et al. and Pires et al. found neral and geranial as the major compounds in samples collected in Santarém‐PA. These same compounds were observed by Prudent' when analyzing the chemical composition of specimens collected in Martinique. This variability can be attributed to factors such as geographic region, climatic conditions, genotypic characteristics, and the time of collection, which influence the biosynthesis and accumulation of secondary metabolites in plants.

### 2.2 Antinociceptive Effect of PeEO Determined by the Hot Plate Test

The central antinociceptive effect of PeEO was assessed using the hot plate test, a well‐established model for evaluating pain responses mediated by central mechanisms, such as those triggered by opioids [19]. This method involves exposing animals to a thermal stimulus by placing them on a metal surface maintained at 55 ± 1°C. The heat induces nociceptive behaviors, primarily paw licking and jumping, which are recognized as supraspinally integrated responses to thermal pain [19, 20].

**FIGURE 1:**
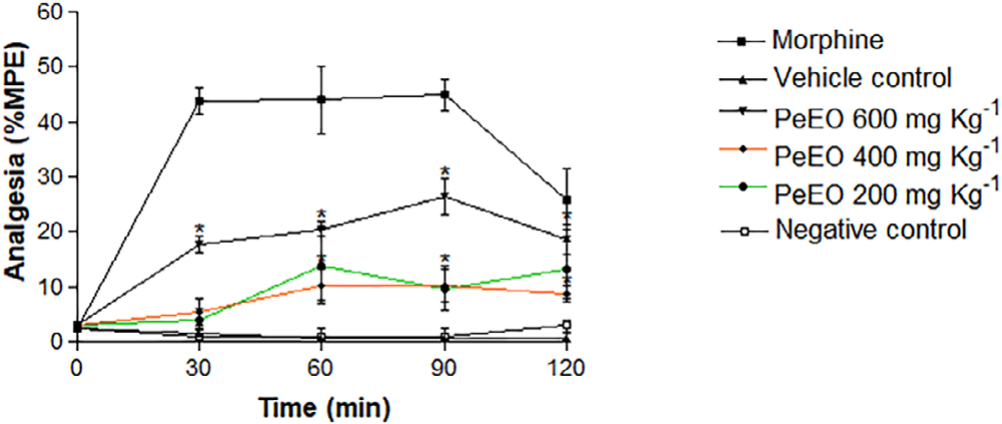
Antinociceptive effect of *Pectis elongata* essential oil (PeEO) in a hot plate test at different time points. Mice were treated with PeEO at doses of 200, 400, and 600 mg/kg (PO) and compared to control groups: positive control (morphine 10 mg/kg, IP), vehicle control (mineral oil), and negative control (distilled water). Latency to nociceptive response was measured at 30, 60, 90, and 120 min after administration. Data are expressed as mean ± SEM (*n* = 6). *p ≤ 0.05 versus negative control. Statistical analysis: one‐way ANOVA followed by Dunnett's Multiple Comparison test.

At this stage, the maximum possible antinociceptive effect (MPE%) was observed for the dose of 600 mg/kg (*p* ≤ 0.05 compared to the negative control). This dose produced a gradual increase in antinociceptive response, starting at 30 min (T30) and peaking at 90 min (T90) after treatment. The 400 mg/kg dose also increased the latency compared to the negative control group (*p* ≤ 0.05), but with less intensity, indicating that intermediate doses can still provide significant antinociceptive relief. In contrast, the 200 mg/kg dose did not produce a significant difference compared to the control group, highlighting the importance of proper dosage for achieving therapeutic effects. The results can be seen in Figure 1.

Our findings are consistent with those reported by Pires et al., who investigated the antinociceptive effect of PeEO using the formalin‐induced nociception model. The authors demonstrated a significant reduction in nociceptive behavior at a dose of 400 mg/kg, in both the inflammatory and neurogenic phases. In contrast, lower doses (200 mg/kg) did not produce relevant effects, suggesting the existence of an effective dose threshold. Notably, in the neurogenic phase, the antinociceptive response observed with 400 mg/kg was comparable to that of morphine, highlighting the therapeutic potential of this natural compound.

The absence of antinociceptive effect at the 200 mg/kg dose may be related to the rapid absorption, metabolism, and excretion of citral, the main component of PeEO. Evidence indicates that, following oral administration in male rats, approximately 50% of the citral dose is eliminated via urine within just 24 h [21]. This pharmacokinetic profile suggests that citral does not remain at the site of action in sufficient concentration or for a long enough period to exert a significant therapeutic effect. Therefore, higher doses may be required to compensate for this accelerated elimination, ensuring that the compound reaches and maintains effective levels in the body to promote the desired antinociceptive activity.

In addition to its pharmacological efficacy, the safety profile of PeEO was also addressed by Pires et al. through acute toxicity testing. The median lethal dose (LD_50_) was estimated to be greater than 2000 mg/kg, which, according to the criteria established by the Organization for Economic Co‐operation and Development [22], classifies PeEO as a substance with low acute toxicity. These findings reinforce the need to investigate the mechanisms of action of PeEO and to optimize dosing strategies to maximize efficacy while minimizing potential adverse effects, providing a robust foundation for future preclinical and clinical research.

Considering that citral represents 95.6% of the composition of PeEO analyzed in the present study, it is plausible that it is the main responsible for the observed antinociceptive activity. However, we cannot rule out the possibility that other constituents, even in smaller proportions, contribute to the effect through synergistic or antagonistic interactions with citral.

Although the mechanism of citral‐induced analgesia is not completely elucidated, studies suggest that it acts by blocking myelinated (Aδ) and unmyelinated (C) nerve fibers, which are excited by inflammatory mediators [23, 24], and desensitizing TRPV1 vanilloid receptors, which play an important role in pain transduction [18]. Stotz et al. [25] demonstrated that citral is a partial antagonist of TRPV1, with potential for the development of anti‐inflammatory and analgesic drugs.

### 2.3 Antinociceptive Mechanisms Observed for PeEO

**FIGURE 2:**
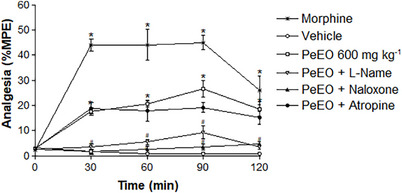
Effect of naloxone, atropine, and L‐NAME on the antinociceptive activity of *Pectis elongata* essential oil (PeEO) in the hot plate test at T30. Mice were pre‐treated intraperitoneally with naloxone (1 mg/kg), atropine (1 mg/kg), or L‐NAME (3 mg/kg) 30 min before administration of PeEO (600 mg/kg, PO) or morphine (10 mg/kg, IP). Nociceptive latency was assessed 30 min after treatment. Data are expressed as mean ± SEM (*n* = 6). *p ≤ 0.05 versus negative control; *p ≤ 0.05 versus PeEO alone. Statistical analysis: one‐way ANOVA followed by Dunnett's Multiple Comparison test.

To determine the underlying mechanism of the antinociceptive effect promoted by PeEO, we evaluated the involvement of the opioid, cholinergic, and nitric oxide (NO) systems in the effects observed after exposure to OE at its effective dose (600 mg/kg). The analysis of the results allowed us to elucidate the involvement of different signaling pathways in mediating the antinociceptive effects promoted by PeEO (Figure 2).

Under our experimental conditions, the antinociceptive effect of PeEO was inhibited after pre‐treatment of the animals with L‐NAME and Naloxone, where a reduction in the effect was observed compared to the values obtained from animals treated only with PeEO (600 mg/kg). These results indicate the involvement of NO and the opioid system in the antinociceptive action of PeEO. These findings are in line with studies that demonstrate the role of NO in antinociception [26]. It is important to highlight that NO can modulate nociception in a complex manner, with effects depending on the dose, experimental model, and the basal level of neuronal excitability [27, 28, 29]. On the other hand, pre‐treatment of the animals with Atropine was not able to block the antinociceptive effect promoted by PeEO, indicating that the cholinergic pathway is not involved in the mechanism of action of this compound in the hot plate test model (Figure 2).

Our results demonstrate that PeEO acts through multiple mechanisms to produce analgesia, involving the opioid and nitrergic systems. These findings indicate that the chemical constituents present in PeEO are promising candidates for the development of new drugs with analgesic potential. The multiplicity of mechanisms may be attributed to the diversity of bioactive substances present in PeEO, with possible synergistic action among the major components in different pain inhibition pathways.

## 3 Conclusions

This study showed that PeEO has antinociceptive activity in mice, particularly at a dose of 600 mg/kg. Chemical analysis revealed that PeEO is rich in citral, a mixture of the monoterpenes geranial and neral, suggesting that this compound is mainly responsible for the observed antinociceptive effect. Experiments with pharmacological blockers indicated that PeEO‐induced antinociception involves the participation of NO and the opioid system, while the cholinergic pathway does not appear to be involved. These findings reinforce the potential of PeEO as a source of new analgesic substances, with the possible advantage of acting through multiple signaling pathways, which may confer greater efficacy and lower risk of tolerance development.

Despite the promising results, this study presents limitations, such as the absence of clinical trials and the lack of isolated evaluation of minor constituents of PeEO, which may contribute synergistically to the observed antinociceptive effect. Future research should investigate the molecular mechanisms involved, identify other bioactive compounds that may enhance its analgesic properties, and evaluate its efficacy and safety in both preclinical and clinical models. These efforts aim to support the development of pharmaceutical formulations based on PeEO, potentially offering new analgesic drugs with greater efficacy and lower toxicity.

## 4 Experimental

### 4.1 Plant Material

Specimens of *P. elongata* were collected in Santarém, Pará, in October 2023, at 9 a.m., during the dry season. The collection was conducted in the cultivation area of local producers (02027'8. 143“ S, 5441.31'. 646” W). The species was identified by Ms. Chieno Suemitsu (UFOPA). The plant's voucher specimen was deposited in the herbarium of the Federal University of Western Pará (UFOPA), under the registration number HSTM‐003432. The specimens were collected following Brazilian laws related to biodiversity protection (SisGen license A192EBE).

### 4.2 EO Extraction

The plant material, previously dried at room temperature (28°C) for 4 days, was crushed and subjected to hydrodistillation in a Clevenger‐type apparatus for 3 h (100 g/L). The obtained oil was dried with anhydrous sodium sulfate, and its content (yield in %) was determined based on the dry weight of the plant, according to the formula described by Santos et al. [30].

### 4.3 Determination of Chemical Composition

The chemical composition of PeEO was determined by gas chromatography coupled with mass spectrometry, using a QP‐2010‐Plus chromatograph (Shimadzu) with a silica capillary column (Rtx‐5 ms, 30 m × 0.25 mm × 0.25 µm). The analysis conditions included: injector temperature at 250°C; oven at 60°C (5 min), with a gradient of 3°C/min, up to 240°C; split injection (1.0 µL in hexane); electron impact ionization at 70 V; ion source temperature at 200°C; and transfer line at 250°C. Helium was used as the carrier gas (1.0 mL/min, 16.5 psi). The identification of components was done by comparing the retention times and mass spectra with standards from [31, 32] libraries.

### 4.4 Animals, Housing Conditions, and Ethical Aspects of the Research

The study used 3‐month‐old male Swiss mice (*Mus musculus*) weighing 25–30 grams. The mice were obtained from the animal nursery of the State University of Pará. The animals were kept in a room with controlled temperature (22 ± 2°C) and a 12 h light/dark cycle, with free access to water and food. To avoid dietary interference with the absorption of substances, the animals were subjected to food restriction 12 h before the experiments, receiving only water. One hour before the start of the tests, the animals were transported to the experimentation room for acclimation. The care and research procedures followed protocol ICBDFBC‐015, following the principles and guidelines of the Brazilian College of Animal Experimentation (COBEA). All experimental procedures were approved by the Ethics Committee on Animal Use of the Federal University of Western Pará (UFOPA), with records filed under No. 0920230266.

### 4.5 Drug and Chemicals

Naloxone, atropine, and L‐NAME were obtained from Sigma (St. Louis, MO, USA). Morphine hydrochloride was obtained from Merck Inc. (Brazil). L‐NAME and atropine were dissolved in saline solution (0.9% NaCl) before use.

### 4.6 Hot Plate Test

#### 4.6.1 Acclimatization Phase

To acclimate mice to the hot plate apparatus, they were initially exposed to the unheated surface of the plate. This procedure is essential to minimize stress and ensure the accuracy of subsequent results [33].

#### 4.6.2 Determination of Effective Dose

To determine the effective dose, the animals were placed on a hot plate maintained at 55 ± 1°C, and the latency to exhibit a nociceptive response (licking, rubbing paws, or jumping) was recorded. To prevent injuries, the maximum time on the hot plate (cut‐off point) was set at 20 s. Baseline latency (control time) was measured once (zero time) before the start of the treatments. The animals (*n* = 6 per group) were treated orally with the PeEO at doses of 200, 400, and 600 mg/kg. Morphine (Sigma—10 mg/kg), water, and mineral oil were used as positive, negative, and solvent control groups, respectively.

Measurements were taken every 30 min, up to 120 min (T30, T60, T90, and T120). The maximum possible antinociceptive effect was calculated using the following formula: MPE% = [observed latency—control latency] / [cut‐off point—control latency] × 100. The dose with the best central analgesic effect (effective dose) was selected to determine the antinociceptive mechanism of action. The experiments were conducted using a double‐blind design, ensuring that both the researchers responsible for administration and those in charge of evaluation had no prior knowledge of the identity of the treatments applied.

#### 4.6.3 Determination of Pain Inhibition Pathways

To determine the pain inhibition pathway mediated by PeEO, the animals (*n* = 6 per group) were pre‐treated by intraperitoneal injection with the following blockers of afferent pain signaling pathways: L‐NAME (3 mg/kg), a selective inhibitor of NO synthase enzyme; Naloxone (1 mg/kg), a non‐selective opioid receptor antagonist; and Atropine (1 mg/kg), a cholinergic receptor antagonist. Animals received PeEO at the effective dose, vehicle, distilled water, or morphine 30 min after the administration of the blockers.

#### 4.6.4 Statistical Analysis

The results were presented as mean ± standard error. Statistical significance was determined by analysis of variance, followed by Dunnett's test. Values of *p* ≤ 0.05 were considered significant differences between groups, with a confidence interval established at 95%.

## Author Contributions

**Angela Palma Amorin**: formal analysis, investigation, and methodology. **Sandra Layse Ferreira Sarrazin**: conceptualization, data curation, formal analysis, funding acquisition, investigation, methodology, project administration, supervision, validation, visualization, writing – original draft, and writing – review and editing. **Leomara Andrade da Silva**: formal analysis, investigation, methodology, visualization, writing – original draft, and writing – review and editing. **Thaiana Neves Coelho**: investigation, methodology. **Adriele Pantoja Cunha**: investigation and methodology. **Vitoria Albuquerque de Lima**: investigation and methodology. **Domingas Machado da Silva**: investigation and methodology. **Pablo Luis Baia Figueiredo**: investigation and methodology. **Ricardo Bezerra de Oliveira**: conceptualization, data curation, formal analysis, funding acquisition, methodology, supervision, writing – original draft, and writing – review and editing.

## Conflicts of Interest

The authors declare no conflicts of interest.

## Data Availability

The authors have nothing to report.
